# Traumatic anterior abdominal wall hernia: A report of three rare cases

**DOI:** 10.4103/0974-2700.76832

**Published:** 2011

**Authors:** Rikki Singal, Usha Dalal, Ashwani Kumar Dalal, Ashok Kumar Attri, Raman Gupta, Anupama Gupta, Bikash Naredi, Deepesh Benjamin Kenwar, Samita Gupta

**Affiliations:** Department of Surgery, Maharishi Markandeshwer Institute of Medical Sciences and Research, Mullana, Ambala, Haryana, India; 1 Government Medical College and Hospital, Sector-32, Chandigarh, India; 2 Adesh Institute of Medical Science and Research, Bathinda, Punjab, India; 3 Department of Anatomy, Adesh Institute of Medical Science and Research, Bathinda, India; 4 Department of -Radiodiagnosis and Imaging, Maharishi Markandeshwer Institute of Medical Sciences and Research, Mullana, Dist-Ambala, Haryana, Punjab, India

**Keywords:** Abdominal wall, blunt trauma, bowel, herniation, management, perforation

## Abstract

Traumatic abdominal wall hernia is a rare condition that can follow any blunt trauma. Associated intra-abdominal injuries are infrequent. In this study, we are reporting three cases, diagnosed as abdominal wall hernia associated with herniation of bowel loops due to blunt trauma. In one case, injury of the herniated bowel was seen. In western medical literature, only few cases have been reported especially with intra-abdominal injuries.

## INTRODUCTION

Traumatic abdominal wall hernias (TAWHs) are extremely uncommon type of abdominal wall hernia as far as the etiology is concerned. Blunt traumatic abdominal hernia is defined as a herniation through disrupted musculature and fascia, without skin penetration with no evidence of a prior hernial defect at the site of injury.[1] Handlebar hernia is an example of traumatic abdominal hernia of anterior abdominal wall which was described by Dimyan *et al*. in 1980.[2] In worldwide literature, less than 50 cases of handlebar hernia have been reported with only three to five cases from India.[3,4] Contrast-enhanced computed tomogram (CECT) and Ultrasonography (USG) can be used to evaluate the associated intra-abdominal injuries. Early surgical repair is necessary for definitive treatment. TAWH as a rare entity has a confusing clinical picture and requires a high index of suspicion for prompt diagnosis and the management. Such hernias, if missed, can result in high morbidity and may prove fatal.

## CASE REPORT

### Case 1

**Figure 1:**
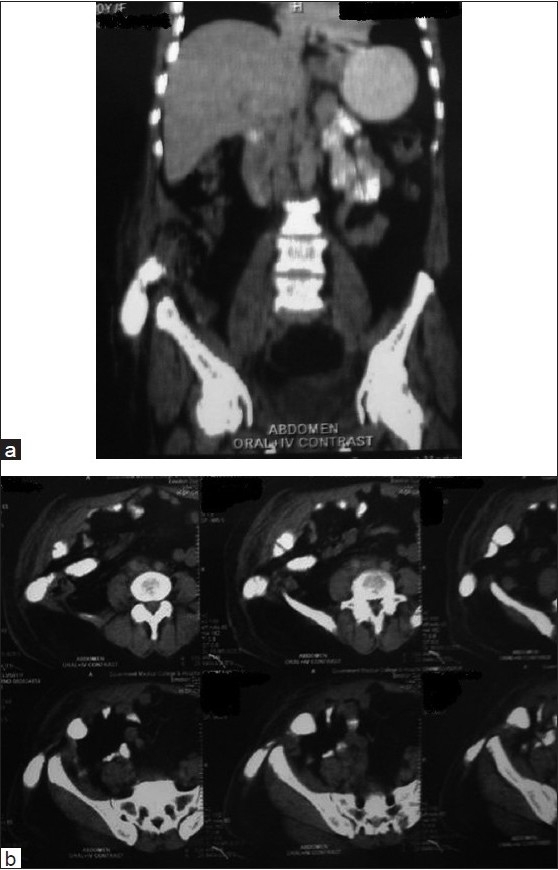
(a and b) Coronal and axial contrast-enhanced sections of the abdomen revealed defect in the anterior abdominal wall with herniation of bowel loops

A 60-year-old female presented with obstipation and vomiting following blunt trauma abdomen in the emergency department 2 days back. Patient had fallen from a bullock cart and had sustained blunt trauma abdomen. There was history of obstipation and vomiting. Vitals were stable. She was obese, and abdomen was moderately distended. There was bruising over the skin in right iliac fossa. Abdominal examination revealed a tender reducible mass of 15 × 20 cm^2^ size in the right iliac fossa. Routine blood investigations were within normal limits. CECT abdomen showed a defect in the anterior abdominal wall above the right iliac crest with herniation of ascending colon and small gut along with avulsed muscle seen in right lumbar region and stranding of subcutaneous fat [Figure 1a and b] There was minimal fluid in the pelvis.

Exploratory laparotomy was performed through an incision over the swelling and findings were a 15-cm wide gap in the aponeurotic part of the external oblique muscle, with small bowel loops along with caecum and ascending colon herniating into the defect. After reduction of herniated contents, abdominal wall defect was repaired primarily with reinforcement by onlay prolene mesh. Patient was discharged in satisfactory condition. On follow-up after 8 months, patient is doing well and asymptomatic.

### Case 2

A 45-year-old male laborer was admitted in emergency department with complaints of pain abdomen and constipation. There was history of fall from a height on to a blunt object. Two days back, the patient was febrile and had tachypnoea and tachycardia. His blood pressure was in normal range. There was abrasion and bruise over the skin, along with surgical emphysema on right lumbar region. On abdominal examination, tenderness and guarding were elicitable in the right lumbar region. Blood tests were normal. USG of the abdomen revealed free fluid in the perihepatic region. Chest X-ray revealed gas under both domes of diaphragm.

**Figure 2:**
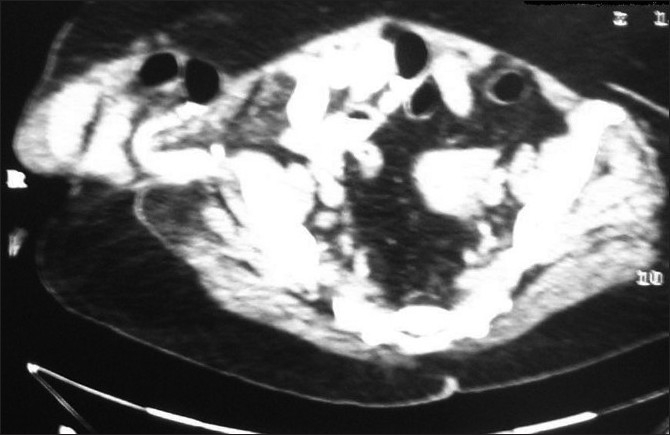
Contrast enhanced computed tomography of the abdomen showed herniation of the bowel loops through the abdominal wall defect into the subcutaneous tissues

CECT of the abdomen showed a defect in the abdominal wall with herniation of small bowel loops [Figure 2]. Rest of the abdominal organs were normal. The abdomen was explored through a midline incision. There was a rent in the right rectus muscle with peritoneal disruption of approximately 8 cm in length. The ileal loops were herniating through the peritoneal defect. A solitary perforation of 2 cm in size was identified approximately 50 cm proximal to ileocaecal junction. Minimal peritoneal contamination was present. Loop illeostomy was done in left iliac fossa with repair of right lumbar defect in layers over a drain. Postoperative period was uneventful. Restoration of bowel continuity was done after 3 months. Patient is doing well in follow-up.

### Case 3

**Figure 3:**
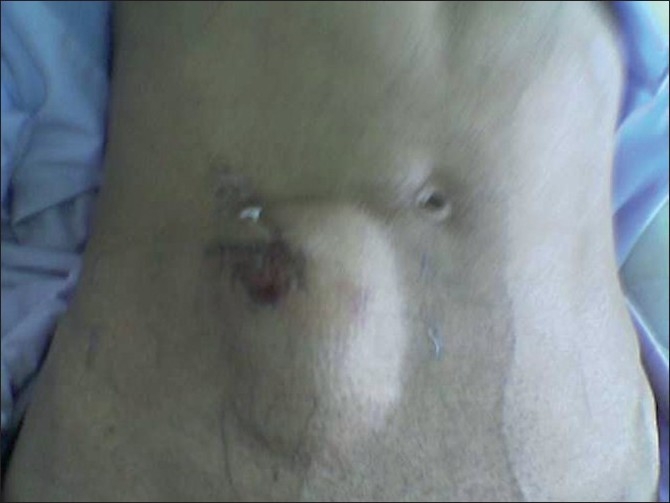
A large swelling seen in right lateral side of the abdomen with post-traumatic contusion

A 45-year-old man, gored by a cow was admitted in emergency department, within 2 h of injury. Patient complained of pain and swelling in the right lower quadrant with localized bruising. Vital signs were stable. On abdominal examination, a tender reducible bulge was present in the right fossa of size 5 × 8 cm^2^ with underlying fascial defect [Figure 3]. Routine blood tests and chest radiography were normal. USG of the abdomen revealed dilated bowel loops herniating through the defect with minimal fluid in the pelvis.

On laparotomy, there was minimal blood stained fluid and a large rent was present in right lower part of the rectus muscle. Ileal loops were reduced, and repair of the mesenteric tear was done. Rest of the intra-abdominal organs were normal. The muscular defects were repaired with absorbable sutures. Postoperatively patient recovered very well. Patient was discharged on 13^th^ postoperative day. On follow-up at 6 months, patient is asymptomatic.

## DISCUSSION

Herniation is a rare occurrence following blunt abdominal trauma. There have been few reports in the literature of trans-rectus herniation. Most herniations are diagnosed at presentation by physical examination or on abdominal CECT, and most authors have advocated immediate laparotomy with repair of the defect because of the high incidence of associated intra-abdominal injury.[5] Traumatic abdominal hernia was first described by Selby in 1906.[6] The criteria for TAWH include immediate appearance of the hernia through the disrupted muscle and fascia after blunt abdominal trauma, and failure of the injury to penetrate the skin, were defined by Clain[7] and Damschen *et al*.[1] It can occur after blunt trauma abdomen which can be classified into low- or high-energy injuries. Low-energy injuries occur after the impact on a small blunt object. High-energy injuries are sustained during motor vehicle accidents or automobile versus pedestrian accidents.[8] The pathophysiology of TAWH involves the application of a blunt force to the abdomen over an area large enough to prevent penetration of the skin; the tangential forces resulting in a pressure-induced disruption of the abdominal wall muscles and fascia, allowing subcutaneous herniation of abdominal viscera through the defect, as proposed by Ganchi.[9] As the skin is more elastic than the other layers of the abdominal wall, it remains intact even though the underlying musculature and fascia are disrupted which gives rise to TAWH.[8,10] In particular, the forces directed tangentially to the abdominal wall can easily produce shearing stresses to the underlying muscles, fascia, and peritoneum. Associated intra-abdominal injuries are infrequent. Damschen *et al*.[1] found that 17 of 28 patients had no associated injury in their review. The other 11 patients had associated injuries, including five in the small intestine (45.5%), three in the colon (27.3%), two in the liver (18.2%), and one in the kidney (9.1%).[11] Stomach rupture, mesocolon, mesenteric hematoma, and cecal deserosation have been reported by other authors.[11] The apparent explanation for the infrequency of associated injury is the commonly observed resistance of hollow viscera to blunt injury and the fact that the trauma delivered in most reported cases is in areas removed from parenchymatous abdominal organs as reported by Yarbrough.[11]

Three types of TAWH were described by Wood *et al*. according to the mechanism and size of injury.[3,12,13] Type I are small defects caused by blunt trauma. In Type II, larger defects occurring during motor vehicle crashes. In Type III, there are abdominal wall defects with bowel loop herniation following deceleration injuries, which are extremely rare.[12,13] Our cases fulfill the criteria of type III, especially the second case which had associated bowel perforation.

The etiology of hernia is usually attributed to congenital, mechanical, and degenerative factors. Blunt traumatic hernias are sufficiently uncommon to preclude identification of specific anatomic patterns, except for the classically recognized pattern of acute diaphragmatic hernia.[14,15] A tender subcutaneous swelling in the abdominal wall is the most common clinical finding with bruising and ecchymosis of the skin. On physical examination, a reducible hernia or swelling with underlying defect may be detected.

CECT and USG of the abdomen are the investigations of choice.[1,11,16] However, CECT is not a reliable investigation to diagnose hollow viscous injury and mesenteric tear. In our second case, CECT diagnosed abdominal wall hernia following blunt trauma but missed associated intestinal perforation, which was found on laparotomy. Once the diagnosis of TAWH is made, some authors advocate early repair both to assess the associated intra-abdominal injuries and to shorten the period of hospitalization and disability. Early repair is considered technically easier. Simple debridement and layered closure of the disrupted musculofacial layers usually have excellent results.

Prompt surgery is required to avoid the complications such as incarceration or strangulation and subsequent morbidity. The incision should be given directly over the traumatic swelling for proper enforcement of the herniated contents and defect.[13,14] The repair of small defects with clear borders is straightforward. In contrast, more prominent disruptions require a variety of factors to be considered, such as the patient’s overall condition, associated intra-abdominal injuries, the defect’s size and site, and available surgical expertise.[14,15,17–19] Primary approximation of the traumatic defect can be done by nonabsorbable sutures with or without mesh, as most case reports indicate.[1,9] Mesh repair is contraindicated in the contaminated wall defects, because of the high risk of mesh infection. TAWHs are uncommon, and it remains controversial whether such patients require urgent laparotomy.

Netto *et al*.[20] carried out a retrospective review of 34 patients with TAWH, and made three recommendations. First, the mechanism of injury should be a deciding factor, whether a patient with TAWH needs an urgent laparotomy or not. Second, clinically apparent anterior abdominal hernias appear to have a high rate of associated injuries and need urgent laparotomy as in one of our cases. Third, occult TAWHs diagnosed only by computed tomography may not require urgent laparotomy or hernia repair as reported.[20] CECT scan is the modality of choice in evaluation of blunt trauma abdomen cases.[21,22,23] It is also useful for identification of the associated injuries. However, we recommend that clinical findings and chest X-ray should be correlated with other investigations. A high index of clinical suspicion is essential, as an accompanying hematoma often confounds the diagnosis.[21]

## CONCLUSION

TAWH should be suspected in a patient with tender, localized swellings of the abdominal wall following blunt trauma. USG and computed tomography of the abdominal are the helpful investigations to diagnose the hernia and associated intra-abdominal injuries. In all cases of wall defects with bowel herniation, one must take up urgent surgical measures to prevent further bowel injury and to avoid complications. Incisions directly over the defects, instead of midline incisions are preferred for proper repair of the defect. Mesh repair is desirable in the elderly with weak anterior abdominal wall so as to prevent the long-term complications of recurrences.
